# Circulating microRNA expression underlying the association of opioid use with low bone mineral density

**DOI:** 10.1093/jbmrpl/ziaf168

**Published:** 2025-10-22

**Authors:** Zannatun Nayema, Jennifer Spillane, Katherine J Motyl, Christine W Lary

**Affiliations:** Roux Institute at Northeastern University, Bouve College of Health Sciences, Portland, ME, 04101, United States; Roux Institute at Northeastern University, Bouve College of Health Sciences, Portland, ME, 04101, United States; MaineHealth Institute for Research, Center for Molecular Medicine, Scarborough, ME, 04074, United States; Roux Institute at Northeastern University, Bouve College of Health Sciences, Portland, ME, 04101, United States; MaineHealth Institute for Research, Center for Molecular Medicine, Scarborough, ME, 04074, United States

**Keywords:** opioids, microRNA, BMD, pathway analysis

## Abstract

Opioid drugs, prescribed for pain management or opioid use disorder, have been associated with decreased BMD and increased fracture risk. Changes in circulating microRNA (miRNA) levels have been observed in opioid-treated patients, and miRNAs are crucial regulators of bone metabolism, but the effects of circulating miRNAs on BMD in the context of opioid use remains unexplored. This study aims to identify circulating miRNAs differentially expressed with opioid use that may explain opioid use effects on BMD. We conducted a cross-sectional analysis of 5692 participants from the Framingham Heart Study Offspring and Third Generation cohorts for which 412 miRNA profiles were obtained via qRT-PCR. BMD measurements were obtained using DXA for most participants, among whom opioid use was reported in 62 (1.1%). We modeled miRNA as a function of opioid use and/or BMD, adjusting for age, sex, and BMI, in linear or logistic regression models. Significant miRNAs associated with both opioid use and BMD were then analyzed using a novel strategy for pathway enrichment to identify biological functions impacted by these miRNAs. We found a significant inverse association between opioid use and BMD after adjusting for covariates (β = −.042, 95% CI = −0.075, −0.007, *p* = .017). We identified 64 miRNAs associated with BMD and 28 miRNAs associated with opioid use (*p* < .05). Ten miRNAs were significantly (*p* < .05) associated with both opioid use and BMD, 9 with opposing effects. Pathway enrichment analysis revealed the involvement of thyrotropin-releasing hormones, phosphatidylserine, vascular endothelial growth factors, integrins, and modulation of calcium and potassium ions. Our study has found preliminary evidence for miRNA-mediated mechanisms by which opioid use impacts bone health, which may guide future translational applications to prevent bone loss in opioid users.

## Graphical Abstract

**Graphical Abstract:**
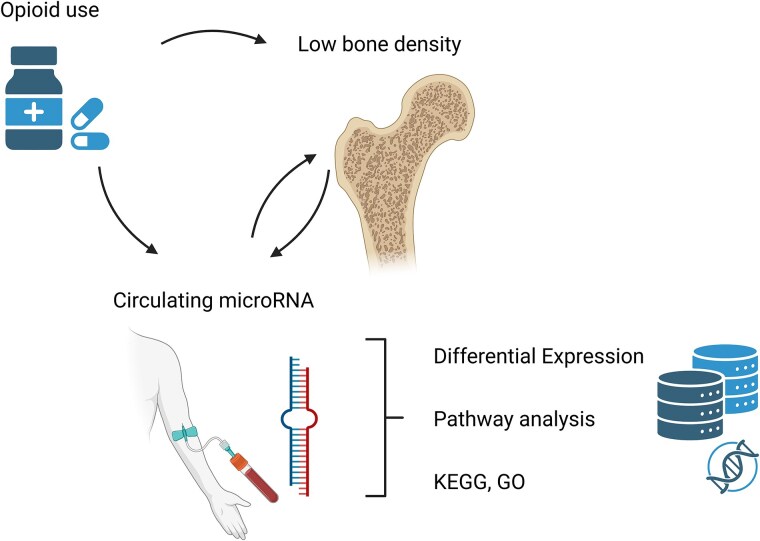

Created in BioRender. Lary, C. (2026) https://BioRender.com/xvf3nka

## Introduction

Opioid use is associated with an increased risk of falls and fractures and decreased BMD.^1–4^ Opioids may impair bone quality^3^ by disrupting bone formation and resorption. Reduced levels of serum osteocalcin, an osteoblast activity marker, have been observed in opioid users.^5^ Morphine stimulates osteoclast production, via opioid and toll-like receptors.^6^ Opioid antagonists appear to aid bone healing.^7^ Indirectly, opioid effects on the central nervous, immune, and endocrine systems may impact bone health, though mechanisms remain unclear.^8–11^

Opioid use has been associated with altered expression of circulating microRNAs (miRNAs) in both humans^12^^,^^13^ and mice.^14^^,^^15^ Furthermore, miRNAs are linked with various skeletal phenotypes and bone metabolism.^16–18^ No human studies to date have explored miRNAs’ association with opioid-related BMD changes, which could offer mechanistic insights. We therefore conducted a cross-sectional analysis of circulating miRNAs associated with opioid use and BMD in participants from the Framingham Heart Study.^19^

## Materials and methods

### Cohort description

Framingham Heart Study is a longitudinal family study of cardiovascular dissease.^19^ A total of 2468 Offspring and 3224 Generation 3 participants had miRNA expression^20^ at exam 8 (2005-2008) and 2 (2008-2011), respectively. Femoral neck BMD^21^ in g/cm^2^ was measured using a GE Lunar Prodigy DXA absorptiometer as previously described.^22^ Medications were Anatomical Therapeutic Chemical coded (Table S1).

### MicroRNA expression profiling

A total of 412 miRNAs were quantified using qRT-PCR via TaqMan, with blood collected after an overnight fast^20^ with absolute difference calculated as change in quantification cycle, |ΔCq| = −(Cq-27).

### Statistical analysis

MicroRNA expression was regressed on opioid use or BMD adjusting for age, sex, and BMI. We used linear or logistic regression based on missingness of miRNAs as performed previously.^16^^,^^17^ If both were significant for a miRNA, a joint model was constructed. Nominal (*p* < .05) and false discovery rate (FDR)-adjusted results (Benjamini–Hochberg) are reported. A sensitivity analysis included smoking, thyroid medication, steroid, and anti-osteoporosis drug use. In a sensitivity analysis, we matched opioid users to non-users (1:2) using Mahalanobis distance based on age, sex, and BMI.

### Pathway enrichment analysis

We adopted a direct enrichment approach^23^ using clusterProfiler^24^ to integrate the Kyoto Encyclopedia of Genes and Genomes (KEGG) pathways^25^ or Gene Ontology (GO)^26^ biological terms. We mapped miRNAs to pathways with a validated target gene(s) from miRecords, miRTarBase, or TarBase using multiMiR,^27^ resulting in 317 out of 412 miRNAs in databases. We used contingency tables for significance testing and performed functional enrichment analysis using QIAGEN IPA (QIAGEN Inc.).^28^

## Results

### Participant characteristics

We included 5692 participants. About 62 (1.2%) used opioids, with oxycodone (39%), tramadol (34%), and dextropropoxyphene (18%) most frequently used (Table 1). Opioid users had higher BMI and a trend towards lower BMD. After adjustment for age, sex, and BMI, opioid use was associated with lower BMD (β = −.042, 95% CI: −0.075, −0.007, *p* = .017).

**Table 1:** Summary statistics for the cohort, including median (interquartile range) for continuous variables and count (frequency) for categorical variables, overall and by opioid use group, with the corresponding *p*-value for difference by opioid use group from a Wilcoxon rank-sum test for continuous variables and chi-squared test or Fisher’s exact test for categorical variables.

**Variable**	**Opioid users** ^**a**^ ***N* = 62**	**Non-users** ^**a**^ ***N* = 5630**	***p*-value** ^**b**^
**Age (years)**	56 (48, 67)	55 (45, 64)	.105
**Sex (female)**	37 (60%)	3017 (54%)	.339
**BMI (kg/m** ^**2**^ **)**	30.5 (24.3, 35.4)	27.3 (24.2, 31.0)	.004
**(Missing)**	0	<11 (<18%)	
**BMD (g/cm** ^**2**^ **)**	0.93 (0.83, 1.03)	0.97 (0.87, 1.07)	.103
**(Missing)**	12 (19.4%)	995 (21.2%)	
**Medication name**			
**Dextropropoxyphene**	11 (18%)		
**Hydromorphone**	<11 (<18%)		
**Morphine**	<11 (<18%)		
**Oxycodone**	24 (39%)		
**Pethidine**	<11 (<18%)		
**Tramadol**	21 (34%)		

^a^Wilcoxon rank-sum test; Pearson’s chi-squared test; Fisher’s exact test.

^b^Median (IQR) or frequency (%).

### Differential expression of miRNAs with BMD and opioid use

**Figure 1:**
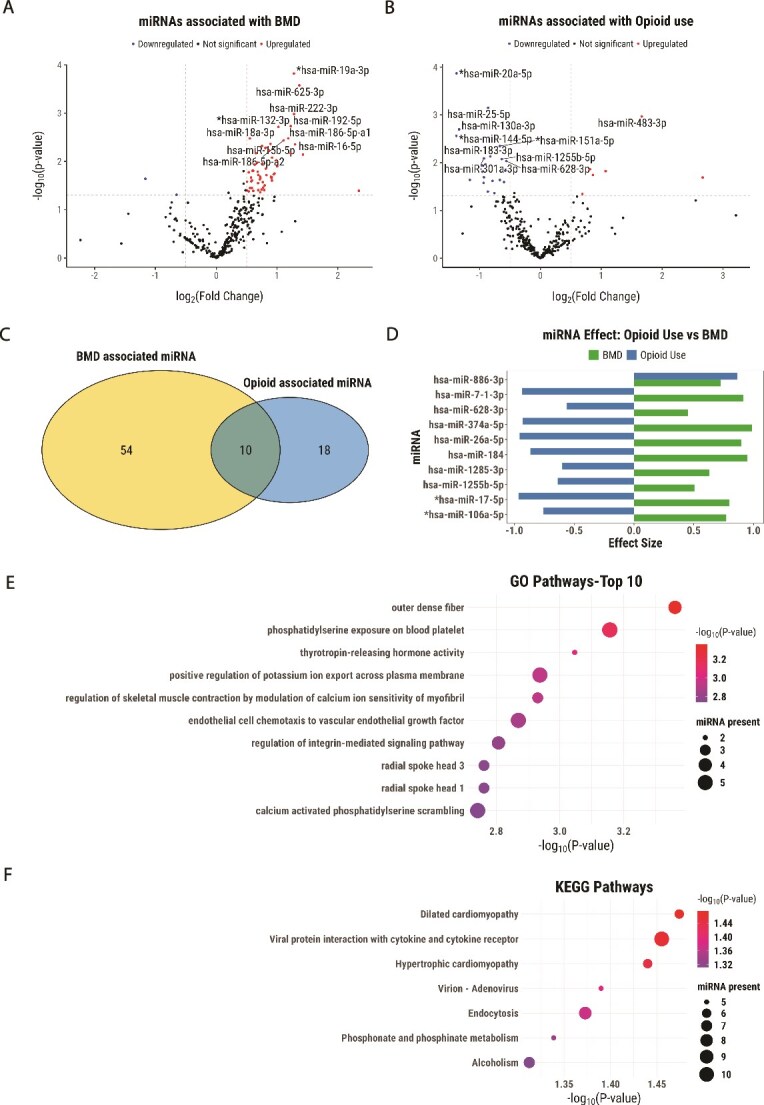
Differential miRNAs associated with BMD and opioid use. Volcano plot showing -log10 (*p* value) vs -log2 (fold change) for differentially expressed miRNAs associated with (A) BMD and (B) opioid use after adjusting for covariates (age, sex, and BMI). Vertical and horizontal dashed lines represent log2 fold change ±0.5 and *p* values <.05, respectively. Red represents upregulation and blue represents downregulation. The top 10 miRNAs are labeled based on the lowest *p*-value and FDR. miRNAs overlapping with those previously reported for association with opioid use^12^^,^^13^ are marked with an asterisk. (C) Venn diagram displaying overlap of the significant (*p* < .05) differentially expressed miRNAs with BMD and opioid use. (D) Bar chart represents the regression coefficient estimate of 10 overlapping miRNAs along the horizontal axis. Green bars represent BMD effects, while the blue bars represent opioid use effects on miRNAs. miRNAs overlapping with those previously reported for association with bone health^16^^,^^40–42^ are marked with an asterisk. Bubble plot showing the enrichment for significant (*p* < .05) (E) GO and (F) KEGG pathways. Each bubble represents a pathway, with the size of the bubble indicating the number of miRNAs associated with that pathway. The color of the bubbles corresponds to the significance level, with lighter colors indicating lower *p*-values or greater statistical significance in the enrichment analysis.

About 64 miRNAs were associated with BMD (*p* < .05), comprising 2 downregulated (3%) and 62 upregulated (97%) (Table S2, Figure 1A). Hsa-miR-654-3p, hsa-miR-486-5p, hsa-miR-19a-3p, hsa-miR-625-3p, and hsa-miR-889 had an FDR value of <0.05.

A total of 30 miRNAs were associated with opioid use (*p* < .05, Table S3, Figure 1B), 6 upregulated and 24 downregulated, with only hsa-miR-20a-5p significant with FDR correction. The sensitivity analysis with matched opioid users and non-users showed similar effect sizes and *p*-values (Table S4).

Ten miRNAs were associated with both opioid use and BMD (Figure 1C, Figure 1D). Except for hsa-miR-886-3p, all were downregulated with opioid use and BMD. Joint model results for these 10 are shown in Table S5 without interaction effects as none were significant. A sensitivity analysis with additional covariates showed similar effect sizes and *p*-values (Table S6).

### Pathway enrichment analysis of miRNAs

Gene Ontology enrichment analysis highlighted 623 terms for 10 differentially expressed miRNAs. Key terms related to bone included thyrotropin-releasing hormone activity (*p* = .0008), endothelial cell chemotaxis to vascular endothelial growth factor (*p* = .001), integrin-mediated signaling pathway (*p* = .001), and calcium-activated phosphatidylserine scrambling (*p* = .001) (Figure 1E). Seven significant (*p* < .05) KEGG pathways were observed (Figure 1F). Ingenuity Pathway Analysis (IPA) identified ribonucleotide reductase signaling pathway (FDR = 0.04).

## Discussion

Our study explored how opioid use may impact BMD via differential expression of circulating miRNAs. Opioid users had significantly lower BMD compared to non-users after correcting for covariates. We identified 10 miRNAs linked to both opioid use and BMD, with 9 downregulated in opioid users and upregulated with higher BMD, suggesting a protective role in maintaining bone health which may be impaired with opioid use. Hsa-miR-17-5p, hsa-miR-106a-5p, and hsa-miR-26a-5p, which were downregulated with opioid use, are previously reported to be differentially expressed with hydrocodone and oxycodone use.^12^^,^^13^ For bone health, hsa-miR-17-5p is crucial for osteoblastic differentiation and cell proliferation,^29^ miR-106a-5p-loaded extracellular vesicles promote bone growth,^30^ and both miRNAs influence stem cell differentiation towards bone.^31^ Finally, miR-26a-5p affects cartilage development and osteogenic differentiation.^32^

Thyrotropin releasing hormone activity, which impacts thyroid stimulating hormone release with opioid use,^33^ has been suggested as a key negative regulator of bone turnover.^34^^,^^35^ Voltage-gated calcium channels and inwardly rectifying potassium channels revealed in our pathway analysis are involved in pain modulation,^36^ opioid addiction,^37^ and osteoblast proliferation and differentiation.^38^ Potential immune mechanisms include morphine effects on immune cell populations,^39^ which may lead to inflammatory bone loss.

The strengths of our study are the large overall sample size, high-quality bone assessments, and sensitivity analyses including a matched design with opioid users and non-users and additional confounders, both showing robustness of conclusions. The limitations are the few opioid users with inability to stratify by opioid type, and the cross-sectional design. Also, we excluded menopausal status as few women were pre-menopausal, none in the opioid group. We didn’t include hormone replacement therapy in women, as it was only present for one opioid user. Physical activity was not included due to missingness. Finally, while we adjusted for multiple testing by reporting FDR, we included nominally significant results in our tables. In conclusion, our study suggests that the differential expression of miRNAs induced by opioid use may influence critical pathways involved in bone metabolism and inflammatory signals associated with bone health.

## Supplementary Material

Supplementary_Tables_to_submit_ziaf168

## Data Availability

Data is made available for this project through an approved data use agreement from dbGAP #33361.
