# Supplementary material for: Circulating microRNA expression underlying the association of opioid use with low bone mineral density
Source: JBMR Plus. 2025 Oct 22;10(1):ziaf168. doi: 10.1093/jbmrpl/ziaf168 (PMC12766910; doi:10.1093/jbmrpl/ziaf168)
Supplement: Supplementary_Tables_to_submit_ziaf168 [file supplementary_tables_to_submit_ziaf168.docx]

**Table S1.** ATC coding for drug medication classes.

| **Variable** | **ATC category** | **Value** |
| --- | --- | --- |
| Opioid user | Pharmacological subgroup | Opioids |
| Thyroid medication user | Chemical subgroup | Thyroid hormones |
| Anti-osteoporosis drug user | Pharmacological subgroup | Drugs affecting bone structure and mineralization |
| Steroid user (glucocorticoids) | ATC code | H02AB07 |

**Table S2. miRNAs significantly associated with BMD after adjusting for covariates.** miRNAs demonstrating statistical significance in BMD model are included in this table. Columns include miRNA, estimate, standard error, p-value, and false discovery rate (FDR). Significant FDR values are boldfaced (all p-values listed are significant).

| **miRNA** | **Estimate** | **SE** | **p-value** | **FDR** |
| --- | --- | --- | --- | --- |
| miR-19a-3p | 1.278 | 0.337 | 0.000 | **0.039** |
| miR-625-3p | 1.367 | 0.375 | 0.000 | **0.039** |
| miR-222-3p | 1.280 | 0.390 | 0.001 | 0.101 |
| miR-132-3p | 1.022 | 0.329 | 0.002 | 0.112 |
| miR-192-5p | 1.226 | 0.394 | 0.002 | 0.112 |
| miR-142-5p | 0.753 | 0.267 | 0.005 | 0.126 |
| miR-15b-5p | 0.891 | 0.312 | 0.004 | 0.126 |
| miR-16-5p | 1.297 | 0.456 | 0.004 | 0.126 |
| miR-186-5p-a1 | 1.185 | 0.404 | 0.003 | 0.126 |
| miR-186-5p-a2 | 1.108 | 0.381 | 0.004 | 0.126 |
| miR-18a-3p | 0.553 | 0.188 | 0.003 | 0.126 |
| miR-19b-3p | 0.809 | 0.290 | 0.005 | 0.127 |
| miR-125a-5p | 0.871 | 0.317 | 0.006 | 0.131 |
| miR-454-3p | 1.225 | 0.453 | 0.007 | 0.131 |
| miR-886-5p | 0.653 | 0.241 | 0.007 | 0.131 |
| rnu44-a1 | 1.426 | 0.531 | 0.007 | 0.131 |
| miR-22-3p | 0.918 | 0.348 | 0.008 | 0.143 |
| miR-374a-5p | 0.988 | 0.381 | 0.010 | 0.154 |
| miR-425-3p | 0.759 | 0.299 | 0.011 | 0.161 |
| miR-576-3p | 0.672 | 0.263 | 0.011 | 0.161 |
| miR-1285-3p | 0.631 | 0.250 | 0.012 | 0.162 |
| let-7g-5p | 0.722 | 0.312 | 0.021 | 0.166 |
| miR-1254 | 0.449 | 0.186 | 0.016 | 0.166 |
| miR-1271-5p-b1 | 0.607 | 0.251 | 0.016 | 0.166 |
| miR-140-3p | 0.815 | 0.332 | 0.014 | 0.166 |
| miR-146b-5p | 0.692 | 0.295 | 0.019 | 0.166 |
| miR-182-5p | 1.002 | 0.402 | 0.013 | 0.166 |
| miR-184 | 0.949 | 0.403 | 0.019 | 0.166 |
| miR-20b-3p | 0.534 | 0.223 | 0.017 | 0.166 |
| miR-20b-5p | 0.726 | 0.318 | 0.022 | 0.166 |
| miR-210 | 0.826 | 0.353 | 0.019 | 0.166 |
| miR-26a-5p | 0.899 | 0.389 | 0.021 | 0.166 |
| miR-29c-3p | 0.911 | 0.399 | 0.022 | 0.166 |
| miR-33a-3p | 0.958 | 0.404 | 0.018 | 0.166 |
| miR-500a-5p | 0.572 | 0.249 | 0.022 | 0.166 |
| miR-502-3p | 0.595 | 0.246 | 0.016 | 0.166 |
| miR-629-3p | 0.681 | 0.284 | 0.016 | 0.166 |
| miR-7-1-3p | 0.913 | 0.396 | 0.021 | 0.166 |
| u6-snrna-a1 | 0.944 | 0.401 | 0.019 | 0.166 |
| miR-183-5p | -1.164 | 0.511 | 0.023 | 0.167 |
| miR-296-5p | 0.716 | 0.316 | 0.024 | 0.167 |
| miR-146a-5p | 0.916 | 0.409 | 0.025 | 0.169 |
| miR-342-3p | 0.587 | 0.261 | 0.025 | 0.169 |
| miR-1255b-5p | 0.506 | 0.228 | 0.026 | 0.170 |
| miR-142-3p | 0.766 | 0.346 | 0.027 | 0.170 |
| miR-197-3p | 0.576 | 0.259 | 0.026 | 0.170 |
| u6-snrna-b | 0.688 | 0.313 | 0.028 | 0.175 |
| miR-628-3p | 0.450 | 0.206 | 0.029 | 0.176 |
| miR-17-5p | 0.797 | 0.370 | 0.032 | 0.187 |
| let-7e-5p | 0.745 | 0.360 | 0.039 | 0.199 |
| miR-103a-3p | 0.691 | 0.336 | 0.040 | 0.199 |
| miR-106a-5p | 0.771 | 0.366 | 0.035 | 0.199 |
| miR-106b-3p | 0.789 | 0.383 | 0.039 | 0.199 |
| miR-1260a | 0.585 | 0.279 | 0.036 | 0.199 |
| miR-29c-5p | 0.481 | 0.234 | 0.040 | 0.199 |
| miR-339-3p | 0.555 | 0.264 | 0.036 | 0.199 |
| miR-345-5p | 0.502 | 0.245 | 0.040 | 0.199 |
| miR-542-3p | 2.348 | 1.146 | 0.041 | 0.199 |
| miR-589-3p | 0.609 | 0.298 | 0.041 | 0.199 |
| miR-625-5p | 0.485 | 0.236 | 0.040 | 0.199 |
| miR-532-5p | 0.533 | 0.262 | 0.042 | 0.201 |
| miR-886-3p | 0.724 | 0.359 | 0.044 | 0.204 |
| miR-484 | 0.200 | 0.100 | 0.045 | 0.207 |
| miR-382-5p | -0.652 | 0.332 | 0.049 | 0.224 |

**Table S3. miRNAs significantly associated with opioid use after adjusting for covariates.** miRNAs demonstrating statistical significance in opioid use model are included in this table. Columns include miRNA, estimate, standard error, p-value, and false discovery rate (FDR). Significant FDR values are boldfaced (all p-values listed are significant).

| **miRNA** | **Estimate** | **SE** | **p-value** | **FDR** |
| --- | --- | --- | --- | --- |
| hsa-miR-20a-5p | -1.381 | 0.362 | 0.000 | **0.039** |
| hsa-miR-25-5p | -0.861 | 0.254 | 0.001 | 0.103 |
| hsa-miR-483-3p | 1.664 | 0.508 | 0.001 | 0.105 |
| hsa-miR-130a-3p | -1.338 | 0.433 | 0.002 | 0.146 |
| hsa-miR-144-5p | -1.383 | 0.462 | 0.003 | 0.160 |
| hsa-miR-151a-5p | -0.669 | 0.236 | 0.005 | 0.218 |
| hsa-miR-183-3p | -0.825 | 0.308 | 0.007 | 0.270 |
| hsa-miR-301a-3p | -0.929 | 0.351 | 0.008 | 0.270 |
| hsa-miR-1255b-5p | -0.637 | 0.242 | 0.008 | 0.270 |
| hsa-miR-628-3p | -0.561 | 0.216 | 0.009 | 0.270 |
| hsa-miR-18a-5p-a1 | -0.905 | 0.356 | 0.011 | 0.276 |
| hsa-miR-17-5p | -0.965 | 0.381 | 0.011 | 0.276 |
| hsa-miR-504 | 0.815 | 0.331 | 0.014 | 0.282 |
| hsa-miR-193a-5p | -0.575 | 0.236 | 0.015 | 0.282 |
| hsa-miR-1274a | 1.072 | 0.441 | 0.015 | 0.282 |
| hsa-miR-26a-5p | -0.956 | 0.395 | 0.016 | 0.282 |
| hsa-miR-886-3p | 0.863 | 0.365 | 0.018 | 0.310 |
| hsa-miR-374a-5p | -0.930 | 0.400 | 0.020 | 0.315 |
| hsa-miR-1304-5p | 2.666 | 1.150 | 0.021 | 0.315 |
| hsa-miR-26b-5p | -1.162 | 0.511 | 0.023 | 0.319 |
| hsa-miR-328 | -0.669 | 0.294 | 0.023 | 0.319 |
| hsa-miR-194-3p | -2.573 | 1.139 | 0.024 | 0.056 |
| hsa-miR-1275 | -0.788 | 0.349 | 0.024 | 0.319 |
| hsa-miR-1285-3p | -0.599 | 0.268 | 0.026 | 0.322 |
| hsa-miR-7-1-3p | -0.934 | 0.421 | 0.027 | 0.322 |
| hsa-miR-184 | -0.865 | 0.422 | 0.041 | 0.455 |
| hsa-miR-505-5p | -0.428 | 0.212 | 0.043 | 0.455 |
| hsa-miR-106a-5p | -0.757 | 0.375 | 0.044 | 0.455 |
| hsa-miR-193b-3p | 0.691 | 0.346 | 0.046 | 0.455 |
| hsa-miR-374a-3p | -1.460 | 0.739 | 0.048 | 0.104 |

**Table S4. miRNAs significantly associated with opioid use in paired analysis in which opioid users are matched to non-users in a 1:2 ratio, with adjustment for covariates.** miRNAs demonstrating statistical significance in opioid use model are included in this table. Columns include miRNA, estimate, standard error, p-value, and false discovery rate (FDR). Significant FDR values are boldfaced (all p-values listed are significant).

|  |  |  |  |  |
| --- | --- | --- | --- | --- |
| \| **miRNA** \| **Estimate** \| **SE** \| **p-val** \| **FDR** \| \| --- \| --- \| --- \| --- \| --- \| \| MIR_20A_5P \| -1.381 \| 0.362 \| 0.0001 \| **0.0393** \| \| MIR_25_5P \| -0.861 \| 0.254 \| 0.0007 \| 0.1033 \| \| MIR_483_3P \| 1.664 \| 0.508 \| 0.0011 \| 0.1050 \| \| MIR_130A_3P \| -1.338 \| 0.433 \| 0.0020 \| 0.1465 \| \| MIR_144_5P \| -1.383 \| 0.462 \| 0.0028 \| 0.1604 \| \| MIR_151A_5P \| -0.669 \| 0.236 \| 0.0045 \| 0.2189 \| \| MIR_183_3P \| -0.825 \| 0.308 \| 0.0074 \| 0.2705 \| \| MIR_301A_3P \| -0.929 \| 0.351 \| 0.0082 \| 0.2705 \| \| MIR_1255B_5P \| -0.637 \| 0.242 \| 0.0084 \| 0.2705 \| \| MIR_628_3P \| -0.561 \| 0.216 \| 0.0093 \| 0.2705 \| \| MIR_18A_5P_A1 \| -0.905 \| 0.356 \| 0.0109 \| 0.2769 \| \| MIR_17_5P \| -0.965 \| 0.381 \| 0.0114 \| 0.2769 \| \| MIR_504 \| 0.815 \| 0.331 \| 0.0138 \| 0.2828 \| \| MIR_193A_5P \| -0.575 \| 0.236 \| 0.0148 \| 0.2828 \| \| MIR_1274A \| 1.072 \| 0.441 \| 0.0151 \| 0.2828 \| \| MIR_26A_5P \| -0.956 \| 0.395 \| 0.0155 \| 0.2828 \| \| MIR_886_3P \| 0.863 \| 0.365 \| 0.0182 \| 0.3113 \| \| MIR_374A_5P \| -0.930 \| 0.400 \| 0.0203 \| 0.3156 \| \| MIR_1304_5P \| 2.666 \| 1.150 \| 0.0206 \| 0.3156 \| \| MIR_26B_5P \| -1.162 \| 0.511 \| 0.0231 \| 0.3199 \| \| MIR_328 \| -0.669 \| 0.294 \| 0.0231 \| 0.3199 \| \| MIR_1275 \| -0.788 \| 0.349 \| 0.0242 \| 0.3199 \| \| MIR_1285_3P \| -0.599 \| 0.268 \| 0.0256 \| 0.3227 \| \| MIR_7_1_3P \| -0.934 \| 0.421 \| 0.0266 \| 0.3227 \| \| MIR_184 \| -0.865 \| 0.422 \| 0.0406 \| 0.4571 \| \| MIR_505_5P \| -0.428 \| 0.212 \| 0.0435 \| 0.4571 \| \| MIR_106A_5P \| -0.757 \| 0.375 \| 0.0437 \| 0.4571 \| \| MIR_193B_3P \| 0.691 \| 0.346 \| 0.0457 \| 0.4571 \| |  |  |  |  |
|  |  |  |  |  |
|  |  |  |  |  |
|  |  |  |  |  |
|  |  |  |  |  |
|  |  |  |  |  |
|  |  |  |  |  |
|  |  |  |  |  |
|  |  |  |  |  |
|  |  |  |  |  |
|  |  |  |  |  |
|  |  |  |  |  |

**Table S5. Significant miRNAs associated with opioid use and BMD.** miRNAs demonstrating statistical significance in both BMD and Opioid use models are included in a joint model in this table without the interaction term (interaction term was not significant in any model). Columns include miRNA, variable, estimate, standard error, p-value, and FDR. Significant p-value or FDR values are boldfaced.

| **miRNA** | **Variable** | **Estimate** | **SE** | **p.value** | **FDR** |
| --- | --- | --- | --- | --- | --- |
| hsa-miR-17-5p | BMD | 0.771 | 0.371 | **0.038** | 0.054 |
| hsa-miR-17-5p | OPIOID_USERS | -0.841 | 0.432 | 0.052 | 0.065 |
| hsa-miR-26a-5p | BMD | 0.864 | 0.389 | **0.026** | 0.042 |
| hsa-miR-26a-5p | OPIOID_USERS | -1.133 | 0.454 | **0.013** | **0.022** |
| hsa-miR-184 | BMD | 0.925 | 0.403 | **0.022** | 0.036 |
| hsa-miR-184 | OPIOID_USERS | -0.772 | 0.476 | 0.105 | 0.121 |
| hsa-miR-106a-5p | BMD | 0.750 | 0.366 | **0.041** | 0.056 |
| hsa-miR-106a-5p | OPIOID_USERS | -0.708 | 0.428 | 0.098 | 0.118 |
| hsa-miR-374a-5p | BMD | 0.963 | 0.381 | **0.012** | 0.021 |
| hsa-miR-374a-5p | OPIOID_USERS | -0.813 | 0.448 | 0.069 | 0.085 |
| hsa-miR-886-3p | BMD | 0.758 | 0.359 | **0.035** | 0.051 |
| hsa-miR-886-3p | OPIOID_USERS | 1.037 | 0.411 | **0.012** | 0.021 |
| hsa-miR-628-3p | BMD | 0.437 | 0.206 | **0.034** | 0.051 |
| hsa-miR-628-3p | OPIOID_USERS | -0.478 | 0.243 | **0.050** | 0.063 |
| hsa-miR-7-1-3p | BMD | 0.884 | 0.397 | **0.026** | **0.042** |
| hsa-miR-7-1-3p | OPIOID_USERS | -0.942 | 0.461 | **0.041** | **0.056** |
| hsa-miR-1255b-5p | BMD | 0.490 | 0.228 | **0.032** | **0.049** |
| hsa-miR-1255b-5p | OPIOID_USERS | -0.528 | 0.265 | **0.046** | 0.062 |
| hsa-miR-1285-3p | BMD | 0.620 | 0.250 | **0.013** | **0.023** |
| hsa-miR-1285-3p | OPIOID_USERS | -0.488 | 0.299 | 0.103 | 0.121 |

**Table S6. Significant miRNAs associated with opioid use and BMD in sensitivity analysis.** miRNAs demonstrating statistical significance in both BMD and Opioid use models are included in a joint model in this table without the interaction term and including current smoking status, thyroid medication use, steroid use, and anti-osteoporosis drug use. Columns include miRNA, variable, estimate, standard error, p-value, and FDR. Significant p-value or FDR values are boldfaced.

| **miRNA** | **Variable** | **Estimate** | **SE** | **p-value** | **FDR** |
| --- | --- | --- | --- | --- | --- |
| MIR_17_5P | BMD | 0.886 | 0.372 | **0.0172** | **0.0421** |
| MIR_17_5P | OPIOID_USERS | -0.823 | 0.432 | 0.0567 | 0.1013 |
| MIR_26A_5P | BMD | 0.972 | 0.391 | **0.0129** | **0.0331** |
| MIR_26A_5P | OPIOID_USERS | -1.156 | 0.454 | **0.0109** | **0.0303** |
| MIR_184 | BMD | 1.016 | 0.405 | **0.0121** | **0.0328** |
| MIR_184 | OPIOID_USERS | -0.772 | 0.476 | 0.1050 | 0.1694 |
| MIR_106A_5P | BMD | 0.799 | 0.368 | **0.0298** | 0.0634 |
| MIR_106A_5P | OPIOID_USERS | -0.690 | 0.428 | 0.1069 | 0.1697 |
| MIR_374A_5P | BMD | 1.111 | 0.382 | **0.0037** | **0.0116** |
| MIR_374A_5P | OPIOID_USERS | -0.838 | 0.447 | 0.0610 | 0.1070 |
| MIR_886_3P | BMD | 0.789 | 0.360 | **0.0286** | 0.0621 |
| MIR_886_3P | OPIOID_USERS | 1.011 | 0.412 | **0.0141** | **0.0353** |
| MIR_628_3P | BMD | 0.484 | 0.207 | **0.0195** | **0.0453** |
| MIR_628_3P | OPIOID_USERS | -0.483 | 0.243 | **0.0474** | 0.0878 |
| MIR_7_1_3P | BMD | 0.935 | 0.398 | **0.0190** | **0.0452** |
| MIR_7_1_3P | OPIOID_USERS | -0.951 | 0.461 | **0.0395** | 0.0811 |
| MIR_1255B_5P | BMD | 0.526 | 0.229 | **0.0216** | **0.0492** |
| MIR_1255B_5P | OPIOID_USERS | -0.528 | 0.265 | **0.0464** | 0.0876 |
| MIR_1285_3P | BMD | 0.658 | 0.251 | **0.0089** | **0.0262** |
| MIR_1285_3P | OPIOID_USERS | -0.516 | 0.299 | 0.0846 | 0.1458 |
